# Educational Case: Essential thrombocythemia

**DOI:** 10.1016/j.acpath.2025.100167

**Published:** 2025-02-28

**Authors:** David T. Danielson, Jeannie M. Muir

**Affiliations:** a Department of Pathology, Walter Reed National Military Medical Center, Bethesda, MD, USA; b Uniformed Services University of the Health Sciences, Bethesda, MD, USA

**Keywords:** Pathology competencies, Organ system pathology, Hematopathology, White cell disorders, Myeloid neoplasia, Essential thrombocythemia, Primary myelofibrosis

## Primary objective

Objective HWC3.2: Myeloid neoplasia. Compare and contrast myelodysplastic syndromes, myeloproliferative neoplasms, and acute myeloid leukemia with respect to morphologic appearance, clinical features, and underlying pathophysiology.

Competency 2: Organ system pathology; Topic: Hematopathology—White cell disorders, lymph nodes, spleen, and thymus (HWC); Learning goal 3: Classification of leukemia and lymphomas.

## Secondary objective

Objective N1.1: Genetic mechanisms of neoplasia. Discuss and provide examples of molecular genetic mechanisms that underlie cancers, including germline mutations, somatic mutations (including point mutations, deletions, ampliﬁcations, and translocations), epigenetic changes, and DNA repair gene effects).

Competency 1: Disease mechanism and processes; Topic: Neoplasia (N); Learning goal 1: Genetic basis of neoplasia.

The following fictional case is intended as a learning tool within the Pathology Competencies for Medical Education (PCME), a set of national standards for teaching pathology. These are divided into three basic competencies: Disease Mechanisms and Processes, Organ SystemPathology, and Diagnostic Medicine and Therapeutic Pathology. For additional information, and a full list of learning objectives for all three competencies, see https://www.journals.elsevier.com/academic-pathology/pathology-competencies-for-medical-education-pcme.^1^

## Patient presentation

The patient is an 81-y-old man who presents for his annual medical visit. His only complaint is increased fatigue over the last 2 y. He reports feeling tired and requiring multiple periods of rest throughout the day. His past medical history includes hypertension, hyperlipidemia, and benign prostatic hyperplasia. He has no prior history of malignancy or chronic inflammatory disorders. He is currently taking rosuvastatin, losartan/hydrochlorothiazide, and a daily multivitamin. He denies smoking and reports occasional alcohol consumption (one to two drinks per week). Review of systems is positive for increased fatigue but otherwise unremarkable.

His heart rate is 61 beats per minute, blood pressure is 146/84 mm Hg, temperature of 97.2°F, and body mass index is 30.95 kg/m^2^. On physical examination, his heart has a regular rate and rhythm without murmurs, rubs, or gallops, his lungs are clear to auscultation bilaterally, and his abdomen is nontender to palpation and nondistended.

A complete blood count (CBC), a comprehensive metabolic panel, and a thyrotrophin level are ordered to evaluate his increased fatigue. His laboratory results show isolated thrombocytosis but are otherwise normal. Given the mildly elevated platelet count, the provider recommends follow-up in 4 mo with repeat CBC for re-evaluation.

## Diagnostic findings, Part 1

The patient's repeat CBC is shown in Table 1. The CBC demonstrates an isolated thrombocytosis with a platelet count of 565 K/UL.

**Table 1:** Complete blood count.

Test	Patient	Reference range and units
WBC	9.1	4.2–9.2 K/UL
HCT	45.7	39.7–50.3 %
HGB	15.2	13.2–16.5 g/dL
RBC	5.00	4.2–9.2 K/UL
MCV	93.2	82.9–99.9 fl
MCH	31.8	27.4–32.7 pg
MCHC	34.1	31.3–35.0 %
RDW	12.5	11.6–14.7 %
Platelet count	565	166–407 K/UL

HCT: hematocrit; HGB: hemoglobin; MCH: mean corpuscular hemoglobin; MCHC: mean corpuscular hemoglobin concentration; MCV: mean corpuscular volume; RBC: red blood cell; RDW: red cell distribution width; WBC: white blood cell.

## Questions/discussion points, Part 1

### What is the differential diagnosis for isolated thrombocytosis?

Thrombocytosis can be either reactive or primary. Reactive causes of thrombocytosis include acute blood loss, infection, chronic inflammatory diseases, malignancy, tissue damage, iron deficiency anemia, and drug reaction.^2^ Primary causes of thrombocytosis include hematologic malignancies and rare inherited conditions.^3^

### What is the mechanism of reactive thrombocytosis?

Platelets play a key role in the inflammatory response by releasing preformed proteins and polypeptides and by synthesizing prostaglandins and leukotrienes. Given these contributions to the inflammatory response, the body has mechanisms to increase platelet production during periods of inflammation. The inflammatory process will lead to increased endogenous levels of inflammatory cytokines. These inflammatory cytokines, specifically interleukin 6, lead to an increased production of thrombopoietin by the liver and kidney.^4^ Thrombopoietin can bind directly to megakaryocytes through *c*-Mpl receptors and leads to megakaryocyte differentiation and proliferation.^5^ Through this mechanism, causes of inflammation, including infection, tissue damage, malignancy, and chronic inflammatory diseases, will lead to thrombocytosis.

Iron deficiency and drug reactions can also lead to a reactive thrombocytosis. Iron plays a key role in regulating lineage commitment in myeloid/erythroid precursor cells. Iron deficiency leads to an increase in megakaryopoiesis, which causes thrombocytosis in some cases of iron deficiency.^6^ While iron deficiency is associated with thrombocytosis, there is typically a concurrent microcytic, hypochromic anemia. Drug-induced thrombocytosis is a relatively rare reaction but can occur through a variety of different mechanisms. Some drugs that are associated with a reactive thrombocytosis include low-molecular-weight heparins, all-trans retinoic acid, antibiotics, corticosteroids, clozapine, epinephrine, gemcitabine, and vinca alkaloids.^2^

### What are the hematologic malignancies associated with thrombocytosis?

Thrombocytosis can be appreciated in both acute and chronic myeloid malignancies. Most commonly, thrombocytosis is seen in myeloproliferative neoplasms (MPN), such as essential thrombocythemia (ET), polycythemia vera (PV), primary myelofibrosis (PMF), and chronic myeloid leukemia (CML), although thrombocytosis can occasionally be seen in specific subtypes of myelodysplastic neoplasms (MDN), myelodysplastic/myeloproliferative neoplasms (MDN/MPN), and acute myeloid leukemia (AML). Essential thrombocythemia, PV, and PMF are clonal disorders, which most commonly stem from mutations in *JAK2*, *CALR*, or *MPL*.^7^ Chronic myeloid leukemia, on the other hand, is characterized by fusion of the Abelson murine leukemia (*ABL*) gene with the breakpoint cluster region (*BCR*), t(9;22) (q34;q11).

MDN/MPNs are neoplasms, which have overlap between MDNs and MPNs. They may present with a combination of cytopenias and cytoses with dysplasia of one or more lineages. Specific subtypes of MDN/MPN can be associated with thrombocytosis, specifically MDS/MPN with *SF3B1* mutation and thrombocytosis and MDS/MPN NOS (unclassifiable).^8^^,^^9^ On the other hand, MDNs are defined by one or more cytopenias in addition to dysplasia in one or more cell lines. Of the MDN subtypes, the only which commonly presents with thrombocytosis is MDN with low blasts and a 5q deletion.^10^ Although uncommon, thrombocytosis can be appreciated in AML. Acute myeloid leukemia is typically defined as having at least 20% blasts in the peripheral blood or bone marrow, although 20% blasts are not required if there are defining genetic abnormalities. Acute myeloid leukemia with *MEDCOM* rearrangement, most frequently inv(3) (q21q26.2) or t(3;3) (q21;q26.2), may be associated with thrombocytosis in some cases.^11^^,^^12^ Rarely, isolated thrombocytosis is due to hereditary conditions. Hereditary thrombocytosis can occur due to mutations of thrombopoietin or mutations in the thrombopoietin receptor gene (*MPL*).^13^

### What further workup should be considered to help narrow the differential diagnosis?

As part of the further workup, the provider may consider ordering an additional CBC and differential count. The additional CBC will help the provider determine whether the cytoses is still present and whether it is stable or worsening. Leukocytes found in peripheral blood include neutrophils, lymphocytes, monocytes, eosinophils, and basophils. A differential count will give the provider an absolute number for each leukocyte in the peripheral blood, which may be increased in MPNs such as CML. In addition, because MPNs are on the differential diagnosis, the provider should consider testing the peripheral blood for mutations in the *JAK2*, *CALR*, and *MPL* genes. Cytogenetic, fluorescence in situ hybridization (FISH), or molecular testing for the BCR-ABL1 fusion must be performed to exclude CML. Testing for inflammatory markers, erythrocyte sedimentation rate (ESR) and C-reactive protein, should be considered. Finally, although unlikely because the patient is not anemic, an iron panel may be obtained to rule out iron deficiency as a cause of the thrombocytosis.

## Diagnostic findings, Part 2

The patient's additional workup is presented in Table 2 (CBC with WBC differential count, iron panel, inflammatory markers, and molecular testing).

**Table 2:** Additional laboratory results.

Test	Patient	Reference range and units
**CBC with Differential**		
WBC	7.9	4.2–9.2 K/UL
HCT	16.1	39.7–50.3 %
HGB	46.9	13.2–16.5 g/dL
RBC	5.06	4.2–9.2 K/UL
Platelet count	545	166–407 K/UL
ABS Neutrophils	4.8	1.9–5.9 K/UL
ABS Lymphocytes	1.9	1.0–2.9 K/UL
ABS Monocytes	0.7	0.3–0.8 K/UL
ABS Eosinophils	0.4	0.0–0.4 K/UL
ABS Basophils	0.1	0.0–0.1 K/UL
**Iron Panel**		
Ferritin	142.5	24–336 ng/mL
Iron saturation	22	20–55%
Total Iron binding capacity	228	228–428 μg/dL
Iron	68	59–158 μg/dL
**Inflammatory Markers**		
ESR	19	7 mg/dL
CRP	7	0–10 mg/dL
**Molecular Testing**		
CALR + JAK2 E12-15 + MPL	POSITIVE: JAK V617F mutation	
BCR-ABL	Not detected	

ABS: absolute; CRP: C-reactive protein; ESR: erythrocyte sedimentation rate; HCT: hematocrit; HGB: hemoglobin; RBC: red blood cell; WBC: white blood cell.

## Questions/discussion points, Part 2

### Summarize the patient's laboratory results

The patient’s repeat CBC and WBC differential count demonstrates an isolated thrombocytosis and a normal WBC count and differential. The iron panel does not demonstrate iron deficiency, and the ESR/CRP is nonelevated. *BCR-ABL* amplification is not detected using reverse transcriptase quantitative polymerase chain reaction (RT-qPCR). A quantitative real-time PCR assay with probes specific to the JAK2 mutant V617F is performed and is positive for detection of the *JAK2* V617F mutation.

### What is the significance of the JAK2 V617F mutation?

JAK2 is a tyrosine kinase, which acts in the cytoplasm to propagate signals from multiple growth factor receptors. The 617 valine on the JAK2 protein is thought to be important for maintaining the JAK2 protein in an inactive conformation. Mutations that lead to phenylalanine in the place of valine at position 617 are thought to increase tyrosine kinase activity by disrupting the inactive conformation of the protein.^14^ The *JAK2* V617F mutation is present in over 90% of PV cases, 65% of PMF cases, and 55% of ET cases.^7^

### How do the above laboratory results change the differential diagnosis? What are the next steps in the workup?

The normal iron panel and lack of anemia help to rule out iron deficiency as a cause of the thrombocytosis. The normal ESR and CRP, no reported clinical history of inflammatory conditions, and minimal systemic systems make a chronic inflammatory condition less likely. Chronic myeloid leukemia is unlikely without leukocytosis and with a negative rtPCR for *BCR-ABL*. Finally, the presence of the *JAK2* V617F and history of isolated thrombocytosis brings ET to the top of the differential, although early PMF is not ruled out. PV is also associated with the *JAK2* V617F mutation; however, the patient does not have a history of erythrocytosis, making PV unlikely. A bone marrow biopsy is necessary to confirm the diagnosis.

## Diagnostic findings, Part 3

**Fig. 1:**
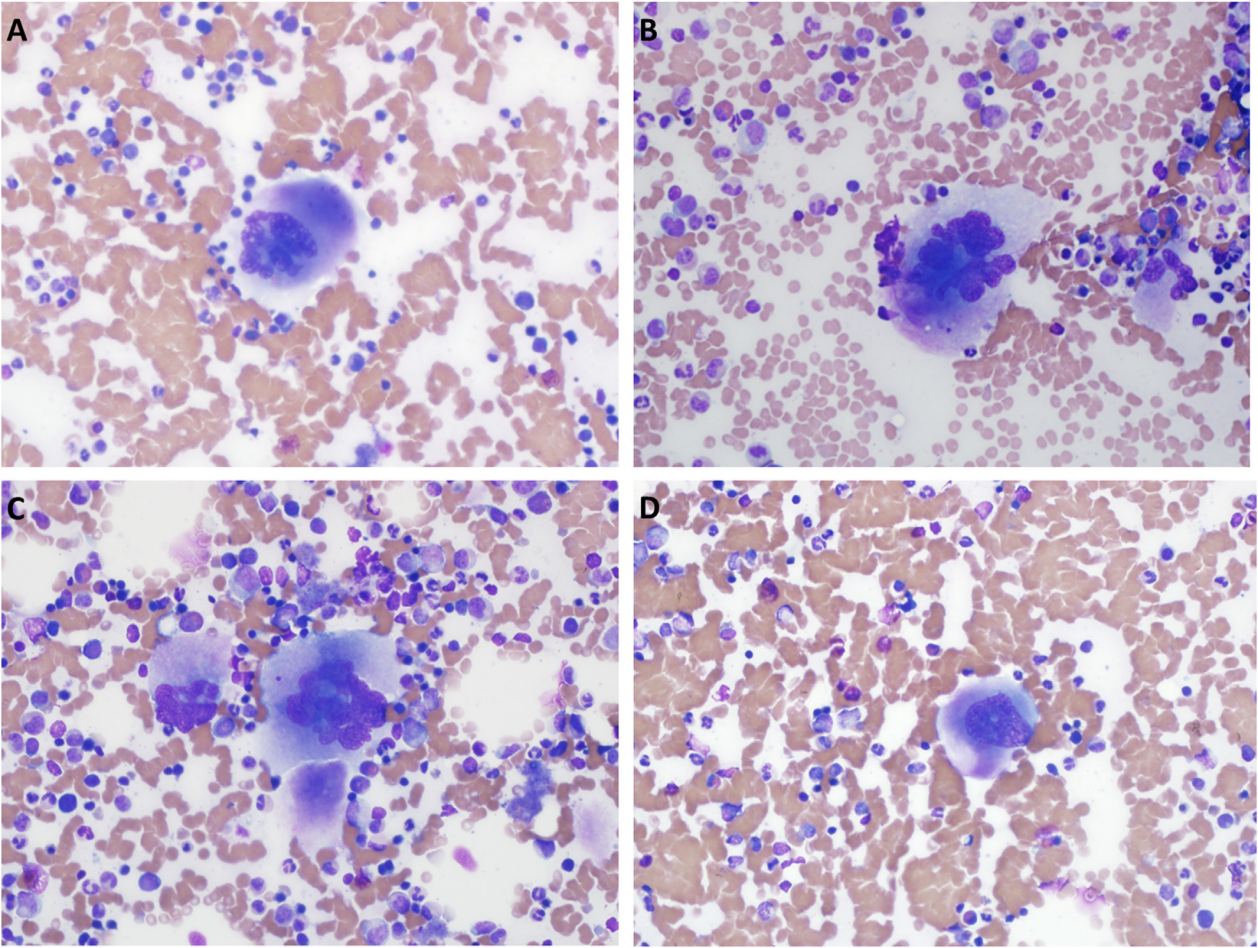
Wright-Giemsa staining of the aspirate demonstrates trilineage hematopoiesis with abnormal megakaryocytes. (A–C) Frequent large and hyperlobated megakaryocytes are present some of which have a vaguely “staghorn” appearing nuclei (original magnification, ×400). (D) Occasional hypolobated megakaryocytes are appreciated (original magnification, ×400).

**Fig. 2:**
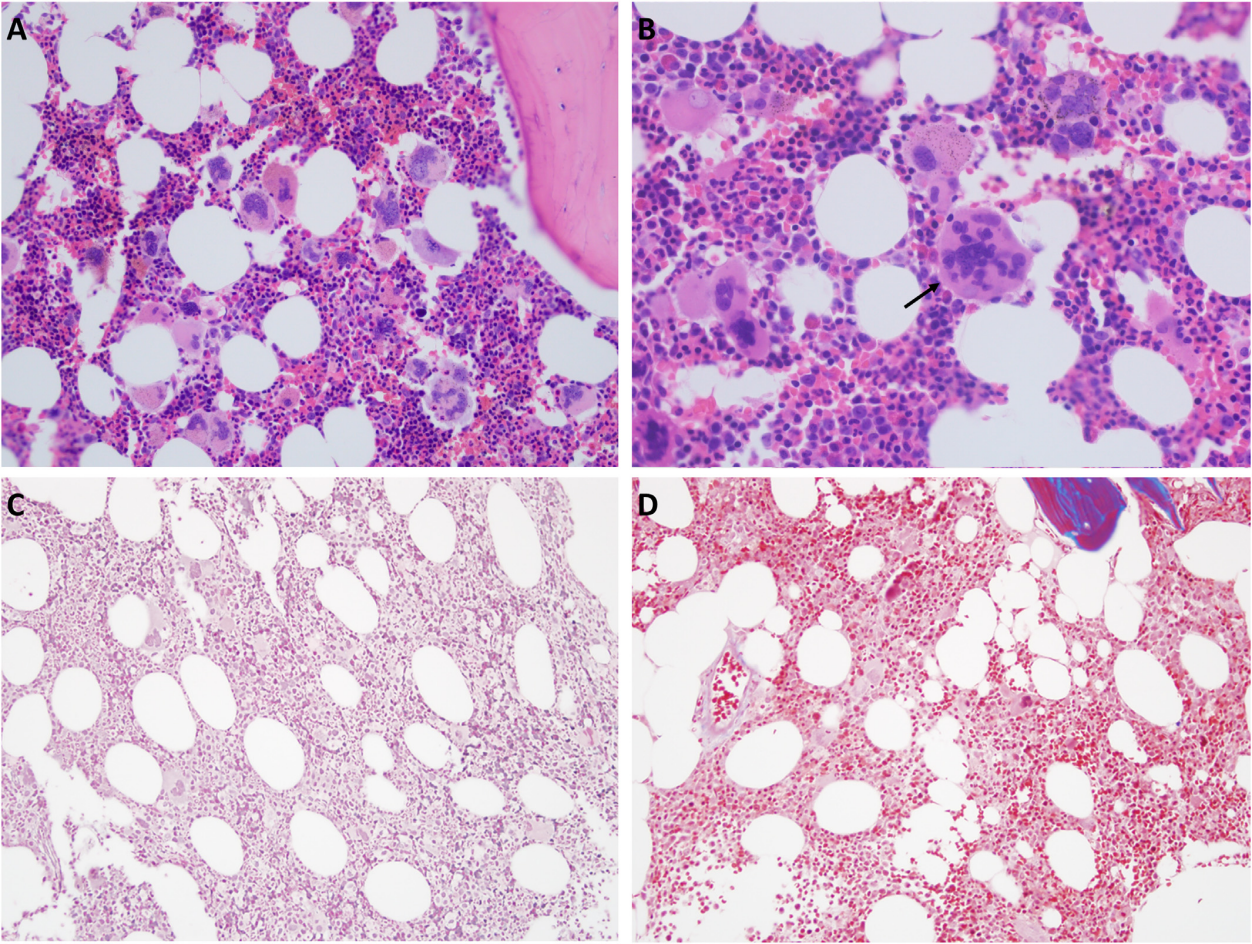
(A) H&E staining of the bone marrow biopsy demonstrates an increase in megakaryocytes, some of which demonstrate hyperlobation. The megakaryocytes are distributed throughout the sample with occasional loose clumping (original magnification, ×200). (B) H&E staining of the bone marrow biopsy demonstrating hyperlobation (arrow; original magnification, ×400. (C) Special staining for reticulin does not demonstrate increased reticulin deposition within the bone marrow (original magnification, ×200). (D) Special staining for collagen (trichrome) does not demonstrate increased collagen deposition within the bone marrow (original magnification, ×200). H&E: hematoxylin and eosin.

A bone marrow aspiration and biopsy are performed by the patient's oncologist. The aspirate and biopsy are subsequently sent to pathology. Wright-Giemsa is used to stain the aspirate. Hematoxylin and eosin (H&E) staining is performed on the bone marrow biopsy along with special staining for collagen and reticulin. Images of the patient's aspirate are shown in Fig. 1, and images of the bone marrow biopsy are shown in Fig. 2.

## Questions/discussion points, Part 3

### How does essential thrombocytosis differ from myelofibrosis histologically?

Histologically, ET commonly presents with normocellular to mildly hypercellular marrow with an increase in megakaryocytes. The megakaryocytes in ET tend to be evenly distributed throughout the biopsy without significant clumping. They are often hypersegmented and enlarged with “staghorn” nuclei. The megakaryocytes in ET do not typically demonstrate significant pleomorphism.^15^

The prefibrotic stage of PMF also presents with increased megakaryocytes histologically. However, the prefibrotic stage of PMF commonly has hypercellular marrow, which may have a left shift in neutrophil granulopoiesis with strikingly abnormal megakaryocytes. The megakaryocytes may be hypersegmented like seen in ET; however, they will typically demonstrate a higher degree of pleomorphism and are often described as “bulbous or cloud-like.” On the bone marrow biopsy of PMF, megakaryocytes are clustered with hyperchromatic and atypical forms.^8^ As PMF progresses to the fibrotic state, there is an expansion in polyclonal fibroblasts, which subsequently leads to increased reticulin and collagen fibrosis. As the marrow becomes increasingly fibrotic, the cellularity will decrease with atypical megakaryocytes becoming more prominent.^15^^,^^16^ Of note, roughly 10% of ET cases will undergo myelofibrotic transformation.^17^ Myelofibrotic transformation of ET is histologically indistinguishable from the fibrotic stage of PMF.^18^

## Diagnostic findings, Part 4

**Fig. 3:**
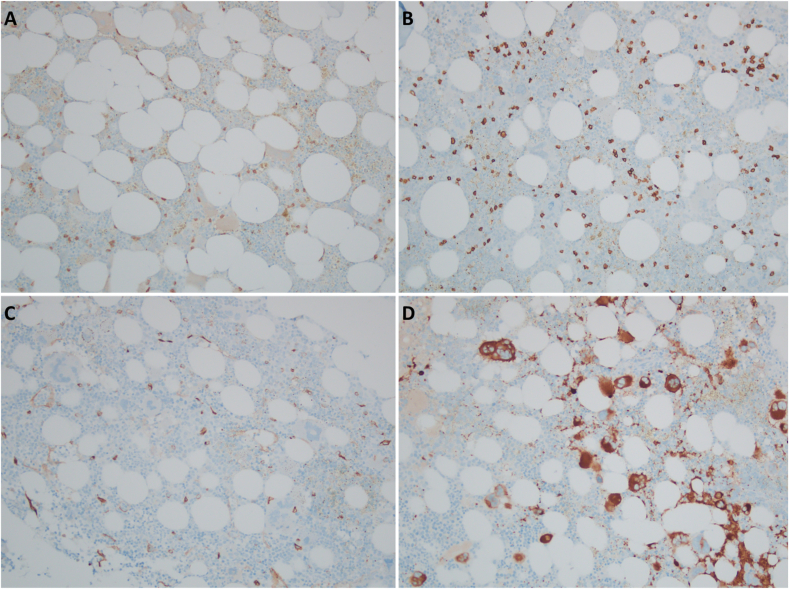
(A) CD3 immunohistochemical stain highlights scattered small T-cells (original magnification, ×200). (B) CD20 immunohistochemical stain highlights scattered small B-cells (original magnification, ×200). (C) CD34 immunohistochemical stain highlights scattered progenitor cells and endothelial cells. The precursor cells make up an estimated 1% of the overall cellularity (original magnification, ×200). (D) CD61 immunohistochemical stain highlights numerous megakaryocytes, which are scattered individually and in loose clusters (original magnification, ×200).

Additional immunohistochemical staining is performed for the following markers: CD3, CD20, CD34, CD61, CD117, and CD138. Representative images from the staining for CD3, CD20, CD34, and CD61 are presented in Fig. 3.

## Questions/discussion points, part 4

### Why were additional immunohistochemical stains performed on this case?

Immunohistochemical stains for CD3, CD20, CD34, CD61, CD117, and CD138 were performed on this bone marrow biopsy to gain further understanding of the cellular composition of the specimen and identify potential underlying pathology. CD3 and CD20 immunohistochemical stains help to evaluate for a lymphoproliferative disorder. CD34 is a marker for hemopoietic progenitors and can be used to assess for a pathology such as an acute leukemia. CD61 stains megakaryocytes and can help visualize megakaryocyte clustering. CD117 (c-kit) is another marker for progenitor cells but also stains mast cells. CD117 staining of progenitor cells would be increased in an acute leukemia, while CD117 staining of mast cells would be increased in a condition such as systemic mastocytosis. CD138 is a marker of plasma cells and would be increased in a plasma cell neoplasm.

### What are the diagnostic criteria for ET?

The World Health Organization (WHO) has four major criteria and one minor criterion for the diagnosis of ET. The major criteria are a platelet count greater than 450 K/μL, bone marrow biopsy showing a proliferation of hyperlobated megakaryocytes with infrequent clusters and no significant fibrosis or left shift, criteria for other neoplasms must not be met (CML, PV, PMF, or other myeloid neoplasms), and presence of the *JAK2*, *CALR*, or *MPL* mutation. The minor criteria for the diagnosis of ET are the presence of a clonal marker (assessed by cytogenetics or sensitive next generation sequencing) and absence of evidence of reactive thrombocytosis. The diagnosis of ET can be made by meeting all four of the major criteria or the first three major criteria plus the minor criteria.^19^ The International Consensus Classification (ICC) follows the same criteria as the WHO for the diagnosis of ET.^20^

The diagnostic criteria for post-ET myelofibrosis have two required criteria and five additional criteria. The two required criteria are a previous diagnosis of ET and bone marrow fibrosis of grades 2–3 (grade 2 on a 0–3 scale and grade 3 on a 0–4 scale). The five additional criteria are anemia with a greater than 2 g/dL decrease in baseline hemoglobin concentration, leukoerythoroblastosis, increasing splenomegaly, elevated lactate dehydrogenase above the reference range, and development of any two constitutional symptoms (defined as >10% weight loss in 6 mo, night sweats, and unexplained fever). To diagnose post-ET myelofibrosis, the two required criteria must be met along with two of the five additional criteria.^19^ Post-ET myelofibrosis diagnostic findings show significant overlap with PMF and without a clear previously diagnosed ET, post-ET myelofibrosis and PMF may be indistinguishable by morphology.

### Why is distinction between ET and PMF important? What is the overall prognosis for ET?

Research findings indicate a notable prognostic difference between ET and PMF. Essential thrombocythemia tends to follow a more benign, indolent course during which patients can remain asymptomatic for extended periods. When complications from ET do arise, they typically involve thromboembolic or hemorrhagic events.^21^ There is a small percentage of ET cases (10%), which will progress to myelofibrosis, and an even smaller percentage (<5%) will progress to the accelerated phase, the blast phase, or myelodysplastic syndrome.^19^^,^^22^^,^^23^ The median survival for ET around 20 y.^21^ Primary myelofibrosis has a significantly worse prognosis, with roughly 14% of patients progressing to the blast phase and a mean survival of around 6 y.^22^^,^^23^

## Teaching Points

• Thrombocytosis can be either reactive or primary.
• Reactive causes of thrombocytosis include causes of inflammation (infection, chronic inflammatory diseases, malignancy, tissue damage), drug reaction, and iron deficiency anemia.
• Primary causes of thrombocytosis include hematologic malignancies and rare inherited conditions.
• The *JAK2* V617F mutation is the most common mutation found in ET followed by *CALR* and *MPL.*
• On histology, ET will present with an increase in abnormal hypersegmented (staghorn) megakaryocytes. The megakaryocytes will be scattered individually with minimal clumping.
• Diagnosis of ET depends on specific criteria as designated by the WHO and ICC.
• Without a clear previously diagnosed ET, differentiation between post-ET myelofibrosis and PMF is difficult due to a near identical histologic appearance.
• ET has a significantly better prognosis compared to PMF with a significantly better overall survival and decreased likelihood of progression to the blast phase.

## Disclaimer

The views expressed herein are those of the authors and do not necessarily reflect the official policy or position of Walter Reed National Military Medical Center, Fort Belvoir Community Hospital, the U.S. Army Medical Department, the U.S. Army Office of the Surgeon General, the Department of the Air Force, the Department of the Army, Department of Defense, the Uniformed Services University of the Health Sciences, or any other agency of the U.S. Government. The identification of specific products or scientific instrumentation is considered an integral part of the scientific endeavor and does not constitute endorsement or implied endorsement on the part of the authors, DoD, or any component agency.

## Funding

The article processing fee for this article was funded by an Open Access Award given by the Society of ‘67, which supports the mission of the Association for Academic Pathology to produce the next generation of outstanding investigators and educational scholars in the field of pathology. This award helps to promote the publication of high-quality original scholarship in *Academic Pathology* by authors at an early stage of academic development.

## Declaration of competing interest

No conflicting relationship exists for any author.
